# Agarwood Induction: Current Developments and Future Perspectives

**DOI:** 10.3389/fpls.2019.00122

**Published:** 2019-02-07

**Authors:** Cheng Seng Tan, Nurulhikma Md Isa, Ismanizan Ismail, Zamri Zainal

**Affiliations:** ^1^Faculty of Science and Technology, School of Biosciences and Biotechnology, Universiti Kebangsaan Malaysia, Bangi, Malaysia; ^2^Institute for Systems Biology (INBIOSIS), Universiti Kebangsaan Malaysia, Bangi, Malaysia

**Keywords:** *Aquilaria*, agarwood, inducer, high-throughput omics, data integration

## Abstract

Agarwood is a resinous part of the non-timber *Aquilaria* tree, which is a highly valuable product for medicine and fragrance purposes. To protect the endangered *Aquilaria* species, mass plantation of *Aquilaria* trees has become a sustainable way in Asian countries to obtain the highly valuable agarwood. As only physiologically triggered *Aquilaria* tree can produce agarwood, effective induction methods are long sought in the agarwood industry. In this paper, we attempt to provide an overview for the past efforts toward the understanding of agarwood formation, the evolvement of induction methods and their further development prospects by integrating it with high-throughput omics approaches.

## Introduction

Agarwood (also known as *gaharu* in the South East Asia, *oud* in the Middle East, *chen xiang* in China, *jinkoh* in Japan and *agar* in India) is a highly valuable aromatic dark resinous heartwood of *Aquilaria* species (Liu Y. Y. et al., 2017). The formation of agarwood is generally associated with the wounding and fungal infection of the *Aquilaria* trees (Liu Y. et al., 2013; Mohamed et al., 2014). The resin is secreted by the trees as defense reaction and deposited around the wounds over the years following the injury, where the accumulation of the volatile compounds eventually forms agarwood (Subasinghe and Hettiarachchi, 2013).

Agarwood has been widely used as therapeutic perfumes, traditional medicine, religious purposes and aromatic food ingredient (Liu Y. et al., 2013). Some of the earliest known uses of agarwood were recorded in ancient literatures, religious scriptures and medical texts. The word “aloes” which means agarwood was found occurring in the Sanskrit poet, Kâlidâsa that can be dated back to c. 4th–5th century CE (Lee and Mohamed, 2016). Meanwhile, the use of agarwood in the prescription of traditional Chinese medicine of the same period had also been recorded. The Chinese medicine uses it as a natural sedative, pain reliever, digestive aid and carminative (Ye et al., 2016; Liu Y. Y. et al., 2017).

Agarwood has high demand throughout the world as a raw material for incense, perfume and medicine purposes, with Middle East and East Asia as the two major regions of consumption (Antonopoulou et al., 2010). As the wealth of the consumer countries has gradually increased in the recent decades, the market’s demand for agarwood started to exceed its supply. Global agarwood prices can be ranging from US$ 20 – 6,000 per kilogram for the wood chips depending on its quality or US$ 10,000 per kilogram for the wood itself (Abdin, 2014). In addition, the value of agarwood essential oil can be as high as US$ 30,000 per kilogram. The annual global market for agarwood has been estimated to be in the range of US$ 6 – 8 billion (Akter et al., 2013), yet a large number of the trades have not been recorded.

*Aquilaria* belongs to the Thymelaeaceae family of angiosperms, which is endemic to the Indomalayan realm. To date, there is a total of 21 *Aquilaria* species which have been documented and 13 of them are recognized as the agarwood-producing species (Lee and Mohamed, 2016). The destructive exploitation of agarwood, however, has badly affected the wild population of all *Aquilaria* species. As a consequence, the genus is now listed as endangered species and protected under Convention on International Trade in Endangered Species of Wild Fauna and Flora (CITES) regulation due to a drastic declination of the species in the wild (Convention On International Trade In Endangered Species [CITES], 2004; Lee and Mohamed, 2016). High demand of quality agarwood in conjunction with the depletion of the wild *Aquilaria* trees implied that the price of the agarwood will continue to soar. As an alternative, mass cultivation and large plantation of *Aquilaria* trees which serve as a sustainable source to obtain agarwood have greatly resolved the shortage of agarwood supply in the global market.

Since healthy *Aquilaria* tree does not form agarwood, leaving it worth next to nothing, the scarcity of naturally occurring agarwood has prompted the development of artificial agarwood-inducing methods. Efforts to artificially induce the agarwood formation can be traced back to as early as 300 C.E. in the Chinese history, where it was recorded that resin deposition accompanied with color changes of internal tissues can happen within a year by injuring the trees (López-Sampson and Page, 2018). Besides mechanical wounding approach, the use of chemical, insect and pathogen-inducing techniques is increasingly common in the agarwood industry nowadays (Liu Y. et al., 2013; Mohamed et al., 2014; Kalita, 2015). All of these induction techniques in any case mimic the natural processes of agarwood formation, which have their own strengths and weaknesses. In this article, we endeavor to provide a more comprehensive coverage of existing induction methods and their development prospects using the advancement of biotechnology. To better understand the agarwood formation process, the molecular mechanism of secondary metabolite biosynthetic pathways underlying the resin production will also be elaborated.

## Agarwood Induction Approaches

The indiscriminate harvesting of agarwood from natural habitats has seriously hampered natural regeneration of *Aquilaria* trees, thus threatening the survival of the species in the wild. In order to meet the high market demand yet to protect the species from extinction, mass plantations of *Aquilaria* trees have been established across the Asian countries to allow sustainable agarwood production (Azren et al., 2018). Since agarwood formation in natural environment is a very long process which can take up to 10 years, the development of effective induction technology has received a great attention as it is extremely crucial to ensure the stability of agarwood yield from the domesticated *Aquilaria* trees.

Naturally, agarwood formation is often linked to the physical wounding or damage of *Aquilaria* trees caused by thunder strike, animal grazing, pest and disease infestations (Rasool and Mohamed, 2016; Wu et al., 2017). These events expose the inner part of the trees toward pathogenic microbes, which elicit the defense mechanism of *Aquilaria* to initiate the resin production. This natural formation process of agarwood has greatly inspired the development of diverse artificial induction methods (Table 1). For example, many traditional induction approaches like nail in setting, holing, burning, trunk breaking and bark removal have adopted the concept of physically wound the trees (Mohamed et al., 2010; Azren et al., 2018). Although it is cost effective and requires only personnel with little or no scientific knowledge on agarwood, but these induction methods usually result in inferior quality and uncertain yield of agarwood.

**Table 1:** Strengths and weaknesses of different types of agarwood inducing methods.

Agarwood formation	Description	Reference
**Natural factors** -Thunder strikes -Broken branches -Animal grazing -Pest and disease -infestations	**Concept** Create wounds for pathogenic microbes to enter and trigger the tree’s defense system **Weaknesses** -Unsustainable, undetermined and extremely low yield -Require extensive and indiscriminately harvesting of wild trees **Advantages** -Possible to obtain high quality agarwood -No cultivation, plantation and induction required	Mohamed et al., 2010; Azren et al., 2018
**Conventional methods** -Physical wounding -Burning-chisel-drilling -Partial-trunk-pruning -Wounding using axe or machete -Bark removal -Cauterizing -Cutting -Holing and nailing	**Concept** -Physical wounds of the tree will trigger the agarwood formation. **Weaknesses** -Laborious -Longer time is required to get the agarwood with uncertain quality -Localized agarwood formation only at the injured areas **Advantages** -Cost effective	Rasool and Mohamed, 2016; Wu et al., 2017
**Non-conventional methods** (1) Biological consortium (Some fungal strain used for induction including *Aspergillus* sp., *Chaetomium* sp., *Fusarium* sp., *Lasiodiplodia* sp., *Penicillium* sp., and *Xylaria* sp.)	**Concept** Introducing microbial cultures into the tree to mimic pathological infection to Aquilaria. **Weaknesses** -Require a long incubation time and localized agarwood formation at the inoculated area -Laborious and time-consuming to make holes and maximized the agarwood yield -Inconsistency of agarwood quality due to different fungal strains or species used **Advantages** - Microbial cultures can be prepared at low cost and easily available - Biological agents are obtained from natural source and often relate to be safe for handling and environmental friendly	Mohamed et al., 2014; Rasool and Mohamed, 2016; Sangareswari Nagajothi et al., 2016
**(2) Chemical inducers** (Phytohormones, salts, minerals, biological-derived substances, and others, e.g., NaCl, H2O2, formic acid, Agar-wit, Agar-bit, and CA-kit)	**Concept** Induce tree’s defense mechanism directly with either chemicals or signaling molecules **Weaknesses** -Skeptical impact on human health and environment -Need to be applied at the right dose to obtain optimal strength of induction **Advantages** -Fast results and high yields -Easy to apply in large-scale plantations -Consistent yield and quality -Can induce agarwood formation in the whole tree/systemic manner	Zhang et al., 2012; Liu X. et al., 2013; Van Thanh et al., 2015

With more understanding on *Aquilaria*-fungal interactions in promoting the agarwood formation, the induction methods gradually shifted from sole mechanical wounding into deliberate wounding coupled with the application of biological inoculum (Jong et al., 2014). Many pure-culture strains of fungi isolated from natural agarwood were found to be effective biological agents to induce agarwood formation in healthy *Aquilaria* trees (Cui et al., 2013; Siburian et al., 2015; Sangareswari Nagajothi et al., 2016). The fungal infected *Aquilaria* trees were reported to deposit agarwood resin around the infected sites as barrier to prevent further fungal intrusion (Cui et al., 2013; Rasool and Mohamed, 2016). One obvious advantage of using fungal inoculum is that it is generally believed to be safe for handling and eco-friendly. However, fungal inoculation will normally give rise to localized and inconsistent quality of agarwood due to the different fungal consortium used. As a solution, laborious holing process and long incubation time is required to maximize the colonized surface area on the tree to produce better quality of agarwood (Mohamed et al., 2014).

Instead of relying on external stimuli to trigger plant responses, either by mechanical wounding or biological inoculum, some induction approaches have been developed to introduce signaling molecules directly and specifically into *Aquilaria* trees to initiate agarwood resin biosynthesis pathways (Liu Y. et al., 2013; Wu et al., 2017). Chemical inducers normally comprise of phytohormones, salts, minerals and biological-derived substances (Zhang et al., 2012; Liu Y. et al., 2013; Van Thanh et al., 2015). Besides, suitable delivery method is often developed together with the chemical formulations to ease the large-scale induction process, such as vessel equipped with transfusion needle (Yang et al., 2014c). To date, several induction approaches have been developed based on the chemical induction concept such as cultivated agarwood kit (CA-kit), the whole-tree agarwood inducing technique (Agar-Wit) and biologically agarwood-inducing technique (Agar-bit). CA-kit is a combined method based on physical wounding and chemical induction, where the inducing agent is applied into the *Aquilaria* tree via an aeration device inserted into the wound (Blanchette and Heuveling, 2009). This method results in satisfying yield and quality, but the procedures are in some way conventional. On the other hands, Agar-Wit is a transpiration-assisted chemical treatment to form an overall wound in the tree, where the preloaded inducer in a transfusion set is distributed via plant transpiration (Liu Y. et al., 2013). Through this method, a larger agarwood coverage area can be achieved, but unfortunately produces more decayed tissues. Similarly, Agar-bit method adopts the idea of distributing the inducing reagent by plant transpiration, except that the reagents are injected directly into the stems of the tree (Wu et al., 2017).

Through chemical induction approach, the time-consuming holing process can be minimized as less induction sites are needed to deliver the inducers throughout the plants via transpiration process. Properly formulated inducer was shown to be able to produce artificial agarwood with quality closely resembled to those obtained from natural source (Liu Y. et al., 2013). In spite of the fast results and high yields, the application of chemical inducers still poses skepticism of toxicity on both human and environment. More assessments on chemical inducers are required to test its effectiveness on fields and also to popularize its use. Chemical inducers are undoubtedly more suitable for mass production with easier quality control than biological inoculum, which is highly potential to substitute conventional induction methods and the use of biological inoculum in agarwood industry.

## The Main Constituents of Agarwood

**FIGURE 1:**
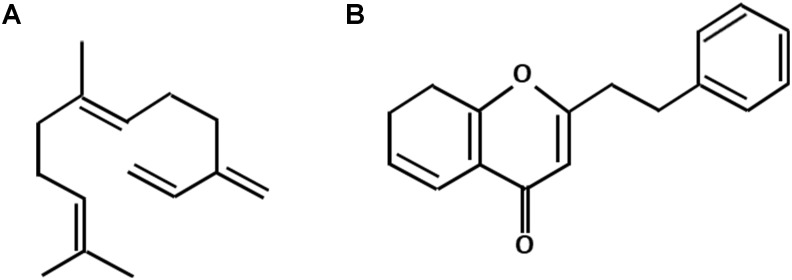
The basic molecular skeleton of sesquiterpenes **(A)** and 2-(2-phenylethyl) chromones **(B)**.

The main attraction of the agarwood industry is its extremely high market value. Yet, the price of agarwood is largely determined by its quality which is graded solely based on human experience from the age-old practices of each country. The unavailability of standard quality grading system can be due to the intricate appearance of the traded agarwood and personal interest. The currently adopted agarwood quality assessment in the market has been extensively reviewed by Liu Y. Y. et al. (2017). Recently, the metabolite analysis of agarwood has gained increasing attention as some studies showed that there is correlation of agarwood quality to its resin yield and metabolite constituents (Pasaribu et al., 2015; Liu Y. Y. et al., 2017). Many studies have been conducted to clarify the metabolite composition of agarwood obtained either from wild or artificially induced methods (Chen et al., 2012; Gao X. et al., 2014; Hashim et al., 2014). It was concluded that the composition of agarwood resin is mainly composed of the mixtures of sesquiterpenes and 2-(2-phenylethyl) chromones (PECs) (Naef, 2011; Chen et al., 2012; Subasinghe and Hettiarachchi, 2015; Figure 1). Meanwhile, the constituents of agarwood essential oil were shown primarily to be sesquiterpenoids (Fazila and Halim, 2012; Hashim et al., 2014; Jayachandran et al., 2014). Together, all of these major compounds and some low abundant volatile aromatic metabolites form the unique and fragrant-smelling property of agarwood.

**FIGURE 2:**
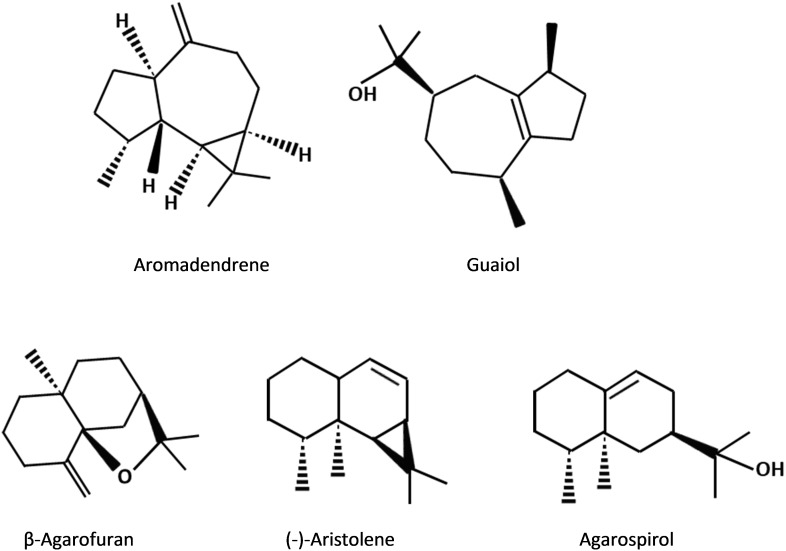
Chemical structures of sesquitepene compounds that commonly exist in agarwood resin.

The number and types of agarwood metabolite constituents of each reported studies vary depending on the agarwood source, extraction methods and analysis approaches used (Fazila and Halim, 2012; Jong et al., 2014; Pasaribu et al., 2015). Nonetheless, there are over 150 compounds as reviewed by Naef (2011) have been identified thus far in agarwood from different sources. Among these compounds, there are 70 sesquiterpenes and about 40 types of PECs which have been recognized in agarwood and their structures have been elucidated (Naef, 2011). Several sesquiterpenes were observed to be more frequently present in agarwood from different studies, including aromadendrene, agarospirol, β-agarofuran, guaiol and (-)-aristolene (Fazila and Halim, 2012; Liu Y. et al., 2013; Jayachandran et al., 2014; Jong et al., 2014; Figure 2). Some sesquiterpenes are reported to be species-specific, such as jinkoh-eremol and *epi*-γ-eudesmol that only present in *A. malaccensis*, while baimuxinal only exists in *A. crassna* and *A. sinensis* (Naef, 2011; Liu Y. et al., 2013; Jong et al., 2014; Hashim et al., 2016). It is worth mentioning that in the study of Pasaribu et al. (2015), the content of aromadendrene was found to be greater in higher grade agarwood and therefore it was suggested as an effective chemical marker for agarwood grading. Besides aromadendrene, Jayachandran et al. (2014) later has proposed an additional marker valencene which can be important in the grading of agarwood oil.

**FIGURE 3:**
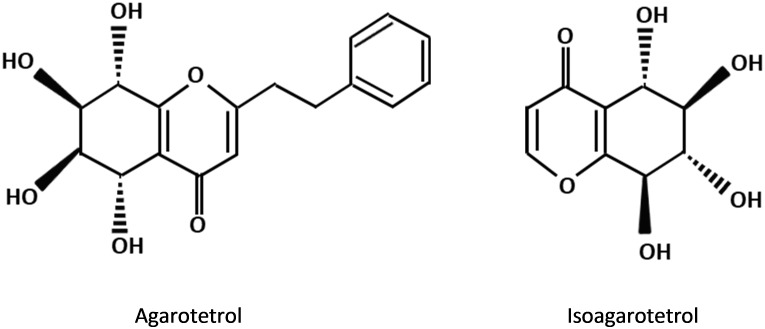
Chemical structures of 2-(2-phenylethyl) chromone derivatives commonly present in agarwood resin.

The PEC derivatives, as other major fragrance constituents of agarwood are the important contributors to the sweet, fruity and long lasting scent of agarwood when it is burnt. These compounds can only be detected by supercritical carbon dioxide and solvent extraction methods but never present in the extract of hydrodistillation (Yoswathana, 2013; Jong et al., 2014). In comparison to the sesquiterpene constituents in agarwood, the types of PECs being determined by GC-MS are relatively limited. Structural studies revealed that all previously reported PECs in agarwood own the same basic skeleton (molecular weight: 250) and similar substituents, i.e., either hydroxy or methoxy groups (Mei et al., 2013). The percentage of 2(2-phenylethyl) chromone and 2-(2-4-methoxy-phenylethyl) chromone in the high grade agarwood such as *kanankoh* can be as high as 66.47 %, which is overwhelmingly higher than the lower-quality agarwood *jinkoh* that has only 1.5% (Ishihara et al., 1993). Furthermore, the presence of certain PEC derivatives in agarwood was proposed to be useful in the evaluation of the grading of agarwood products (Shimada et al., 1982). There are 17 types of chromone derivatives which are agarwood specific and potential marker for the purpose of authentication (Naef, 2011). The substituted chromones, such as agarotetrol and isoagarotetrol (Figure 3), were shown to have positive correlation with the quality of agarwood obtained in the market with some exceptions (Shimada et al., 1986).

The types and derivatives of major compounds in agarwood are extremely wide and diverse, indicating the miscellaneous fragrance properties of agarwood from different species and regional sources. The better insight of agarwood metabolites will definitely facilitate the identification of universally accepted biomarkers for agarwood grading. Since the publication of the comprehensive review of Naef (2011) regarding the major constituents of agarwood, new compounds continue to be discovered in the later studies (Wu et al., 2012a; Yang et al., 2014b; Wang et al., 2015). The number of discovered compounds in agarwood will certainly be further increased in the future.

## The Biosynthesis Pathways of Agarwood Constituents

Agarwood formation can be related to the self-defense mechanism of *Aquilaria* trees in response to biotic and abiotic stresses (Gao et al., 2012b; Singh and Sharma, 2015). Stresses trigger the defense responses of *Aquilaria* species which in turn initiate the secondary metabolite biosynthesis and the accumulation of agarwood resin. Previously, we have mentioned that sesquiterpenes and PEC derivatives are the main constituents in agarwood. Hence, it is crucially important to understand the metabolic pathway for the regulation and biosynthesis of sesquiterpenes and chromone derivatives in *Aquilaria* species to effectively induce the agarwood formation.

**FIGURE 4:**
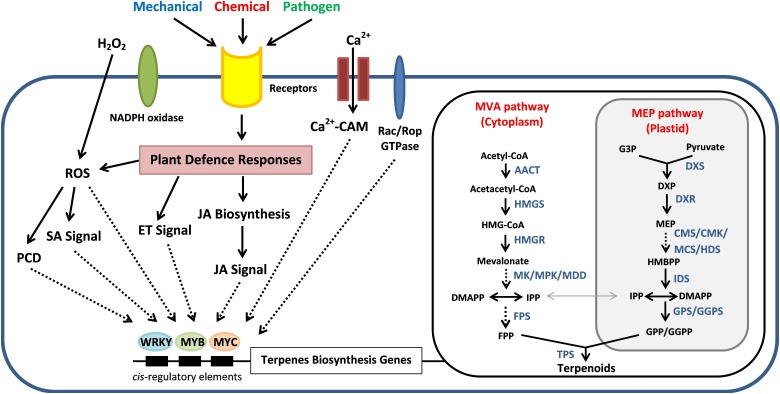
Schematic relationships between the wound-induced signal transduction mechanisms for the sesquiterpene biosynthesis and regulation in *Aquilaria* species for the agarwood production. External stimuli trigger the Ca^2+^ signaling pathway and induce the defense responses of *Aquilaria* species via hydrogen peroxide (H_2_O_2_) pathway, ethylene (ET) signals, Jasmonic acid (JA) signals, and salicylic acid (SA) signals. MeJA treatment triggers H_2_O_2_ production that can induce programmed cell death (PCD) and increase the sesquiterpene synthesis. These signaling molecules activate the transcription factors such as MYB, MYC, and WRKY, which will bind to the *cis*-element on the promoter of terpenes biosynthesis genes in the mevalonic acid (MVA) and methylerythritol phosphate (MEP) pathways and also the downstream terpene synthase genes (*TP*s). Direct and indirect interactions are shown as solid and dotted lines, respectively. *AACT*, acetyl-CoA C-acetyl transferase; *HMGS*, hydroxymethylglutaryl (HMG)-CoA synthase; *HMGR*, HMG-CoA reductase; *MK*, mevalonate kinase; *MPK*, phosphomevalonate kinase; *MDD*, mevalonate diphosphate decarboxylase; *DXP*, 1-deoxy-D-xylulose 5-phosphate; *DXS*, DXP synthase; *DXR*, DXP reductoisomerase; *CMK*, 4-(cytidine 50-diphospho)-2-C-methyl-D-erythritol kinase; *MCS*, 2-C-methyl-D-D-erythritol-2,4-cyclo diphosphate synthase; *HDS*, (E)-4-hydroxy-3-methylbut-2-enyl diphosphate synthase; *IDS*, isopentenyl diphosphate synthase; *GPS*, geranyl disphosphate synthase; *GGPS*, geranylgeranyl diphosphate synthase.

In plants, the isoprenoid precursors for the biosynthesis of sesquiterpenes, triterpenes and sterols has generally been assumed to be provided from the mevalonic acid (MVA) pathway in cytosol. In plastids, the 1-deoxy-D-xylulose-5-phosphate (DXP) or known as methylerythritol phosphate (MEP) pathway provides precursors for the production of monoterpenes, diterpenes, and carotenoids (Rohmer, 1999; Dong et al., 2015; Singh and Sharma, 2015). These two pathways biosynthesise C5 homoallylic isoprenoid precursor, that is isopentenyl pyrophosphate (IPP) and its electrophilic allylic isomer dimethylallyl pyrophosphate (DMAPP). An exchange of IPP and DMAPP was observed to happen in between plastids and cytosol even with the spatial partitioning of the two pathways (Dong et al., 2015). The production of IPP and DMAPP precursors from pyruvate and acetyl-CoA involves a series of enzymes according to the respective pathway (Figure 4). The genes encode for these enzymes have been identified from *Aquilaria* species through transcriptome sequencing analysis (Xu et al., 2013; Ye et al., 2016). These C5 isoprene units will later be channeled into the generation of C15 farnesyl pyrophosphate (FPP) by sequential condensation reactions in the presence of FPP synthase (FPS) (Rohmer, 1999; Yang et al., 2013; Ye et al., 2016). The FPS is one of the key-limiting enzymes responsible for the sesquiterpene biosynthesis (Gaffe et al., 2000; Yang et al., 2013; Liu X. M.et al., 2017). The genes encode for FPS have been cloned from *Aquilaria microcarpa* (*Am-FaPS-1*) (Kenmotsu et al., 2011) and *Aquilaria sinensis* (*AsFPS1*) (Yang et al., 2013). The transcript level of *AsFPS1* was reported to be higher in stem and roots than the leaves, suggesting that sesquiterpene synthesis in *Aquilaria* species tends to be tissue-specific. Besides, the expression of *Am-FaPS-1* was shown to be up-regulated upon exposure to methyl jasmonate (MeJA), yeast extract and Ca^2+^-ionophore A23187, indicating that the two former chemicals are effective to initiate the sesquiterpene biosynthesis pathway whereas Ca^2+^ can act as signaling molecule during the activation process (Kenmotsu et al., 2011). This provides clues for the artificial induction of agarwood formation via exogenous chemically induced approaches by triggering the sesquiterpene biosynthetic pathway in *Aquilaria* trees.

In the final stage of sesquiterpenes production, the enzymes accountable for the diversification of sesquiterpene mainly come from the classes of sesquiterpene synthases (SesTPs) and cytochrome P450 dependent mono-oxygenases (P450s). The SesTP enzymes are responsible to catalyze the formation of multicyclic scaffold complexes from FPP, followed by oxidative functionalization of the resulting scaffolds by cytochrome P450 enzymes. The added hydroxyl groups by P450s can serve as molecular handles for further modifications, such as alkylations, esterifications and the addition of sugar residues (Pateraki et al., 2015). In addition, the P450 enzymes which carry out stereospecific hydroxylation on the hydrocarbon backbones, that is important for the novel chiralities and further modifications of the sesquiterpene molecules, have never been reported from *Aquilaria* species thus far. Similarly, the NADPH-dependent cytochrome P450 oxidoreductases (POR) in *Aquilaria* that act as redox partners of P450s catalysis activity are basically unexplored. Several studies have been reported to isolate genes encode for SesTPs from *Aquilaria*, which can be considered as the early attempts to study SesTPs involved in the agarwood formation (Kumeta and Ito, 2010; Xu et al., 2013). In the study of Kumeta and Ito (2010), five genes encode for sesquiterpene synthases which shared highly similar amino acid sequences have been isolated from *Aquilaria crassna*. Three out of these genes have been successfully expressed in *Escherichia coli* and enzymatically converted FPP into δ-guaiene as their major product. Besides, three other sesquiterpene synthase genes (*ASS1*, *ASS2*, and *ASS3*) identified from *A. sinensis* via transcriptome sequencing have been revealed to encode enzymes that produce δ-guaiene as well (Xu et al., 2013). The isolation of *SesTP* genes was also described in *A. malaccensis* where the temporal and spatial expression of the two *SesTP*s reported in the study, i.e., the guaiene (*AmGuaiS1*) and sesquiterpene synthase (*AmSesTPS1*), was elucidated (Azzarina et al., 2016). The *AmSesTPS1* was found to be highly expressed after 6 h of wounding while *AmGuaiS1* was induced after 2 h of wounding at a magnitude of 18- and 5.5-fold higher than unwounded control, respectively. Recently, a novel sesquiterpene synthase gene (*As-sesTPS*) was isolated from *A. sinensis* where the recombinant As-sesTPS catalyzed FPP into nerolidol (Ye et al., 2018). Expression analysis showed that the transcript level of *As-sesTPS* was much higher in agarwood than the healthy wood, implying that the gene can be participated in the agarwood formation. Despite the fact that many sesquiterpene compounds have been discovered from the agarwood, the corresponding SesTPs responsible for their production have yet to be reported from *Aquilaria*. For that reason, the sesquiterpene biosynthesis pathways involve in the functionalization of terpenes in *Aquilaria* is urged for further clarification.

On the other hand, chromones are a large group of secondary metabolites with wide-ranging potential therapeutic indications toward immunomodulation, inflammation, cancer, diabetes, neurological conditions, bacterial and viral infections (Khadem and Marles, 2011; Yang et al., 2012; Tawfik et al., 2014). Chromone is derived from a polycyclic organic compound namely benzopyran ring, with a keto group substitution on its oxime ring. It is generally believed that derivations of chromones take place as a consequence of the convergence of multiple secondary metabolite biosynthetic pathways involving pentaketide pathway, shikimic acid pathway and the addition of nitrogenous moiety from amino acids or other sources (Khadem and Marles, 2011). Owing to the extensive pharmacological properties associated with its bicyclic ring structure, chromones have been used as the privileged scaffold in the development of new drugs (Reis et al., 2017). The PECs are small class of chromones, which hold a phenylethyl substituent at the C_2_ of benzopyran ring of the chromone that happened to be structurally unique in the family (Ibrahim and Mohamed, 2015). Until now, the PECs have only been found to be present in a few species of plants for example *Bothriochloa ischaemum* (Wang et al., 2001), *Imperata cylindrical* (Liu X. et al., 2013), *Cucumis melo* L. (Ibrahim, 2014), *Gyrinops salicifolia* (Shao et al., 2016), and *Aquilaria* species (Wu et al., 2012b; Yang et al., 2014a). Recently, a hypothetical scheme for the biosynthetic pathway of PECs was proposed by Liao et al. (2018) based on in-dept analysis of agarwood chemical constituents using GC-EL-MS and UPLC-ESI-MS/MS methods. In their study, the PECs was found to be the major agarwood resin constituents, which is comprised mostly of flindersia-type 2-(2-phenylethyl) chromones (FTPECs). The formation of FTPECs is further elucidated to be possibly catalyzed by type III polyketide synthase (PKs) through condensation of dihydro-cinnamoyl-CoA analogs and malonyl-CoA with 2-hydroxy-benzoyl-CoA to produce PEC scaffold that will subsequently be catalyzed by hydroxylases or *O*-methyltransferases (OMTs) to form structurally diverse FTPECs (Liao et al., 2018). Recent study showed that salinity stress could induce the biosynthesis of PECs in *A. sinensis* calli (Wang et al., 2016). Transcriptomic analysis of these salt-induced *A. sinensis* calli have identified several upregulated candidate genes potentially involved in the biosynthesis of PECs, including three OMT-encoding genes (flavonol-OMT 1, flavonol-3-OMT and caffeoyl-CoA-OMT) and a type III polyketide synthase gene encodes for chalcone synthase 1 (AsCHS1).

In spite of the recent progress made on the understandings of PECs biosynthesis, a tremendous effort is necessary to experimentally determine the missing steps in this complex PEC biosynthetic pathway. Even with the inadequate knowledge on the detailed PEC biosynthetic pathway, artificial synthesis of chromones and some of its derivatives is nevertheless feasible due to the advancement of chemical processes (Goel and Makrandi, 2006; Tawfik et al., 2014). Agarwood is a rich source of PEC derivatives which deserves further investigation to uncover the structure of new chromone compounds and improve the understanding toward its biosynthetic mechanism at the molecular level.

## Signaling and Regulation Mechanism of Agarwood Formation

In nature, the probability of getting agarwood-containing *Aquilaria* trees are extremely low (1–2%), where can only be found on pathogenically infected or wounded trees (Cui et al., 2013; Chhipa and Kaushik, 2017). Therefore, it is sensible to assume that there is a wound-inducible signal transduction process causing the expression of sesquiterpene synthases prior to agarwood formation. In order to clarify the relationship of wound signal transduction and regulation of agarwood formation, high-throughput studies on agarwood formation have recently gained attention in researches (Table 2). A schematic diagram of the proposed signal transduction mechanism of sesquiterpene biosynthesis and regulation in *Aquilaria* species is provided in Figure 4.

**Table 2:** Selected publications in signaling and regulation of agarwood formation.

Year	Description	Reference
2012	Identification of conserved and novel microRNAs in *Aquilaria sinensis* based on small RNA sequencing and transcriptome sequence data	Gao et al., 2012b
2013	Identification of genes related to agarwood formation: transcriptome analysis of healthy and wounded tissues of *A. sinensis*	Xu et al., 2013
2014	Profiling of microRNAs under wound treatment in *A. sinensis* to identify possible microRNAs involved in agarwood formation	Gao Z. H. et al., 2014
2015	Hydrogen peroxide promotes programmed cell death and salicylic acid accumulation during the induced production of sesquiterpenes in cultured cell suspensions of *A. sinensis*	Liu et al., 2015
2015	Cloning, expression and characterization of COI1 gene (AsCOI1) from *A. sinensis* (Lour.) Gilg	Liao et al., 2015
2016	Transcriptome sequencing of chemically induced *A. sinensis* to identify genes related to agarwood formation	Ye et al., 2016
2016	Jasmonic acid is a crucial signal transducer in heat shock induced sesquiterpene formation in *A. sinensis*	Xu et al., 2016
2016	Salinity stress induces the production of 2-(2-phenylethyl)chromones and regulates novel classes of responsive genes involved in signal transduction in *A. sinensis* calli	Wang et al., 2016
2017	Transcription factor AsMYC2 controls the jasmonate-responsive expression of ASS1 regulating sesquiterpene biosynthesis in *A, sinensis* (Lour.) Gilg	Xu C. et al., 2017

The mitogen-activated protein kinase (MAPK) signaling pathway has been proposed as wound-induced signaling mechanism for the agarwood formation in *A. sinensis*, which phosphorylates downstream transcription factors (TFs) like MYB or WRKY that eventually lead to the expression of sesquiterpene synthase genes (*ASS*s) (Xu et al., 2013). The MAPK signaling cascade consists of three sequentially activated components [MAPK kinase kinases (MAPKKKs), MAPK kinases (MAPKKs), and MAPKs], which is highly conserved signaling mechanism in eukaryotes in mediating extracellular signals to downstream responsive genes (Sinha et al., 2011; Xu C. et al., 2017). Xu et al. (2013) has reported that a total of 41 unigenes from the transcriptome analysis of wounded *A. sinensis* are annotated as being related to MAPK signaling pathway and 25 to calcium signaling pathways which may play roles in wound-induced agarwood formation. In plants, calcium ions (Ca^2+^) are important intracellular secondary messenger molecules to regulate many signal transduction pathways reacting to the external stimuli (Tuteja and Mahajan, 2007). Earlier studies in other plants have shown that TFs are crucial regulators in stress-responsive signaling pathways to transmit signals to different cellular centers to activate plant adaptation/defense mechanisms against adverse environments, including TFs like bZIP, ERF, EIN3, MYB, MYC, and WRKY (Ambawat et al., 2013; Phukan et al., 2016; Schmiesing et al., 2016). Overexpression of AaWRKY1 in *Artemisia annua* was found to have positively regulated the expression of amorpha-4,11-diene synthase gene (*ADS*) and significantly increased the production of artemisinin (Ma et al., 2009). The expression of cotton TF GaWRKY was also shown to upregulate the sesquiterpene synthase genes for the biosynthesis of (+)-δ-cadinene and gossypol sesquiterpene (Xu et al., 2004).

Besides mechanical wounding, the MeJA is an effective elicitor to increase the sesquiterpenes content in *Aquilaria* (Xu et al., 2013, 2016; Xu Y. H. et al., 2017). Previous studies have shown that heat shock can increase the expression of genes involved in the Jasmonic acid (JA) biosynthesis, including allene oxide cyclase (*AOC*), allene oxide synthase (*AOS*), lipoxygenase (*LOX*) and 12-oxophytodienoate reductase 3 (*OPR3*) genes, which subsequently lead to the production of JA and the accumulation of sesquiterpene in the *A. sinensis* suspension cell culture (Xu et al., 2016). The *A. sinensis* coronatine-insensitive protein 1 (AsCOI1), acts as a receptor in MeJA signaling pathway, has been cloned and characterized (Liao et al., 2015). Expression study of *AsCOI1* has demonstrated that the gene was expressed in a tissue-specific pattern which is highest in stem, followed by root and leaves. The findings imply that resin production due to external stimuli may be more responsive in stem of *Aquilaria*. The *AsCOI1* gene is responsive toward early treatment of MeJA, mechanical wounding and heat stress. The application of MeJA in *A. sinensis* has increased the expression level of 17 wound signaling-related genes, including TFs *WRKY4* and *MYB4*, protein kinases *CAPK*s, *MAPK*s and *MAPKK*, NADPH oxidase *noxB* and some regulators related to signal molecules MeJA, ethylene and hydrogen peroxide (Xu et al., 2013). Interestingly, the hydrogen peroxide (H_2_O_2_) producing NADPH oxidase noxB was found to be significantly up-regulated by the MeJA treatment (Xu et al., 2013; Gong et al., 2017), which is consistent with the findings that MeJA triggers H_2_O_2_ production in plants (Orozco-Cardenas et al., 2001; Hung et al., 2006). A study on suspension cultures of *A. sinensis* revealed that H_2_O_2_ can induce the programmed cell death (PCD) and sesquiterpene synthesis by the elevated expression of *ASS* genes due to the endogenous accumulation of salicylic acid (SA) (Liu et al., 2015). Meanwhile, the expression of jasmonate-responsive key sesquiterpene synthase *ASS1* in *A. sinensis* was described to be regulated by a TF AsMYC2 (Xu Y. H. et al., 2017). As an immediate-early responsive gene toward MeJA treatment, AsMYC2 binds to the *ASS1* promoter containing a G-box motif to initiate the expression of *ASS1*. Similarly, the homolog of AsMYC2 in *Arabidopsis* (MYC2) was shown to be MeJA-responsive and up-regulated the expression of two sesquiterpene synthases (*TPS11* and *TPS21*) upon treatment (Hong et al., 2012). In addition, MeJA treatment has successfully induced the synthesis of 3 sesquiterpenes, that are α-guaiene, α-humulene and δ-guaiene in *A. crassna* cell culture (Ito et al., 2005; Kumeta and Ito, 2010).

In contrary to the sesquiterpene biosynthesis pathway, the biosynthesis and regulation of PECs remains almost unknown. In the study of Wang et al. (2016), forty-one PECs have been produced in *A. sinensi*s calli by salt treatment. With the help of high-throughput transcriptome analysis, a total of 18,069 differentially expressed transcripts between the control and the NaCl-treated *A. sinensis* calli induced by 24 or 120 h salinity stress were obtained. Many differentially expressed genes are reported to be involved in the hormone signal transduction; including genes encode for MAPK cascades, receptor-like kinases, Ca^2+^ signal transduction and transcription factors (Wang et al., 2016).

Nonetheless, the pace of research did not just stop at searching for effective inducers and their underlying signal transduction process, but also extended to their post-transcriptional regulation mechanism. By deep sequencing of sRNAs from healthy control and wounded samples of *A. sinensis*, Gao et al. (2012a) have identified ten stress-responsive miRNAs from 74 putative conserved miRNAs and their hairpin forming precursors were also confirmed. Expression pattern revealed that six of these stress-responsive miRNAs were up-regulated, including miR159, miR168, miR171, miR396, miR397, and miR408, whereas miR160 and miR398 were down-regulated and continued their reduced level at 2 day point (Gao et al., 2012a). The different responses of miRNAs reacted toward treatment and the effect lasted for various time lengths reflecting the diversity of their positions in the post-transcriptional regulation of wound response in *A. sinensis*. Among the identified miRNAs, the down-regulated miR398 is of interest where it was demonstrated to negatively regulate the pathogen-associated molecular pattern (PAMP)-triggered callose deposition and plant innate immunity against bacteria (Li et al., 2010). The oppositely reacted miR160 and miR398 in *A. sinensis* suggested that they might be important regulators and play a more distinct role on agarwood formation.

Further study on miRNAs profiling of wounded *A. sinensis* showed that some of the most conserved miRNAs such as miR159 and miR396 families elevated and subsided quickly in early period of treatment implying their function at the upstream of wound responses (Gao Z. H. et al., 2014). The down-regulated miR396b2 in wounded tissues of *A. sinensis* was suggested to involve in the biosynthesis and accumulation of agarwood constituents. The target of miR396b2, which has glutamyl-tRNA reductase activity, was believed to bind NADP and produce NADPH. The NADPH is the cofactor for the two key enzymes in terpene biosynthesis, i.e., 1-deoxy-D-xylulose 5 phosphate (DXR) and 3-hydroxy-3-methylglutaryl-CoA (HMGR) (Nagegowda, 2010). Furthermore, plant P450s that oxidatively functionalize the terpene scaffolds also require reducing agents for its catalytic activity, which is commonly provided by NADH or NADPH. Despite in the situation of lacking sequenced genome, high-throughput transcriptome analysis provides a feasible approach to examine the overall changes of gene expression on *Aquilaria* species responding to a variety of stresses. Further investigation of the functions of the identified regulator sequences would help to reveal the regulation mechanism of agarwood formation.

## Future Prospects of Agarwood Induction Technology

Previous studies have shown that agarwood formation can be influenced by many factors. Together with the exceedingly complex agarwood resin composition, it is believed that agarwood formation is an intricate process which involved a variety of physiological changes occurs on *Aquilaria* trees to cope with the external stimuli either in the form of biotic or abiotic. This whole agarwood formation process is in any case inseparable from the gene-expression response of the trees toward the triggering factors. Consequently, future improvement of agarwood induction technology should emphasize on two aspects that are to further improve the induction efficiency and to screen more responsive lines of *Aquilaria* for resin production under breeding program.

In order to improve the induction efficiency, an induction technique plays a decisive role. The concept of currently available induction approaches can be summarized as either to provide external stimuli to activate the production of plant signaling molecules that eventually lead to the resin biosynthesis, or to bypass the external stimuli via direct introduction of signaling molecules to the plants. In any case, the overall concern is to increase agarwood yield and quality as well to reduce human intervention (e.g., holing process) during the induction process. Since the aforementioned physical wounding and biological induction method have their inevitable drawbacks of inconsistent agarwood quality and requiring intensive workforce, the chemical induction method can be regarded as a promising approach for further optimization (Table 1). Comprehensive understanding of agarwood formation at the molecular level via high-throughput using omics approach such as trancriptomic and metabolomic appears to be advantageous for more targeted and directional improvement of the induction formulation rather than the trial- and error-based experimentations.

By coupling with omics approaches such as single molecule real time sequencing technology (SMRT) which offers longer read lengths and highly contiguous *de novo* assemblies (Rhoads and Au, 2015), thus it tends to be particularly useful for unsolved problems in genome and transcriptome of non-model *Aquilaria* species where their genomes are not available. With longer reads, the highly repetitive non-coding regulatory sequence of genes controlling the agarwood resin production can be easily obtained. Sequence similarity/identity-based integration can be used to establish the cross-reference data sets between the query sequences and their homologous references from various public databases and genetic resources, where genomic sequence structures, domain features, promoter regions and gene ontology for motifs can be assigned (Mochida and Shinozaki, 2011). The development of sequencing technology has made the future research on the whole genome sequencing of *Aquilaria* species to be easier to fill in the lack of genome-wide information in the current situation. Data integration based on genome sequence is important to allow analysis of global changes of transcriptome through whole genome microarrays. The gene expression analysis of induced *Aquilaria* at its entirety can be examined in broad coordinated trends by this approach, which is indiscernible by individual assays. The expression profiles obtained in this way will help to identify potential agarwood-producing biomarker genes that are important indicators for downstream applications of agarwood induction.

**FIGURE 5:**
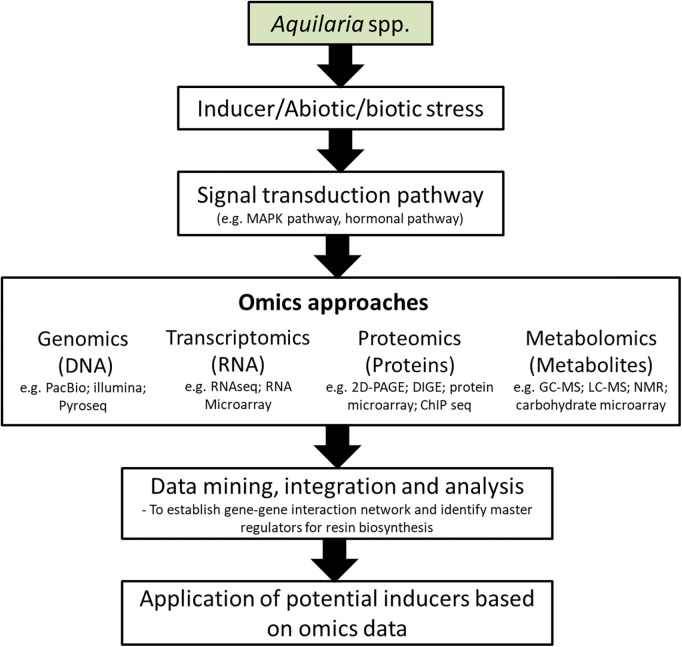
Schematic representation of application of omic approaches in the development of agarwood inducers. PacBio, Pacific Biosciences; Illumina, Illumina sequencing; Pyroseq, pyrosequencing; RNAseq, RNA sequencing; 2D-PAGE, 2-dimensional polyacrylamide gel electrophoresis; DIGE, differential gel electrophoresis; ChIP seq, chromatin immunoprecipitation sequencing; GC-MS, gas chromatography-mass spectrometry; LC-MS, liquid chromatography-mass spectrometry; NMR, nuclear magnetic resonance.

The integration of systems biology and omics approaches, covering genomics, transcriptomics, proteomics, metabolomics and functional analysis; provide a potential solution to comprehend the multigenic nature of resin biosynthesis in *Aquilaria*. On the basis of large number of previously conducted agarwood induction experiments, transcriptomic and metabolites studies (Naef, 2011; Gao X. et al., 2014; Ye et al., 2016; Wu et al., 2017), integrated omics analysis can indeed serve as a platform to build a more comprehensive picture of agarwood resin biosynthetic mechanism that involve different omics layers. The development of high-throughput mass spectrometry (MS), microarray and sequencing technologies (DNA and RNA) have made it possible to integrate such data into a system biological framework via integrated-omics which would help to predict gene-gene interactions, identify driver genes and the molecular signatures of agarwood formation (Figure 5). Likewise, potential signaling molecules for agarwood formation can be predicted in a wake of this dramatically increased availability of data. Experimentally validated effective signaling molecules can be added into the existing recipe of chemical inducer to further enhance the induction performance. Moreover, predetermination of resin composition or the agarwood quality is conceivable with deep understanding of the key quality indicators and the specific pathways involved for agarwood production by integrated elucidation of different metabolite and transcript profiles under different induction methods. Future improvement of agarwood induction efficiency should accompany with the development of monitoring system for early detection of non-responsive trees which could avoid cutting down of *Aquilaria* trees that are unsuccessfully been induced. This can be achieved by monitoring the expression of a set of genes involved in agarwood resin biosynthesis.

Aside of the effective inducer, the responsiveness of *Aquilaria* trees toward stimulation is another determining factor for the production of agarwood. By knowing that the degree of plant response on stimuli is largely dependent on their genetic makeup, the utilization of highly responsive *Aquilaria* line as induction target is expected to further increase the agarwood yield rather than optimizing the inducer recipe alone. Conventionally, selective breeding based on phenotypic selection have been adopted to develop new plant lines with desirable traits. Current technology associates these beneficial traits of plants to genetic (DNA/RNA variations) or biochemical (signature metabolites) markers to allow marker-assisted selection (MAS). The approach of MAS offers a great promise for the selection of elite *Aquilaria* lines as these biomarkers can be applied to predict the phenotypic characteristics before these features develop into more noticeable. Such biomarkers can also be used for the development of fast and targeted diagnostic assays that will assist the selection program. As an alternative to obtain a high-yield line, a combined approach of genetic engineering (e.g., CRISPR-Cas9 genome editing technology) with tissue culture could pose a possibility to manipulate the key regulator genes of *Aquilaria* involved in the agarwood production which will help to fine tune or redirect the metabolic flux toward desired metabolic pathways. On the whole, an integrated and high-throughput strategy will provide sufficient information to continually improve the agarwood induction methods, which is superior compare to the traditional way of induction method establishment that rely on the visual observation and personal experience. A deeper insight into the essential compounds and the biosynthesis mechanism of agarwood resin would greatly ease to control the stability of agarwood yield, quality and its price in the future.

## Author Contributions

CT, NI, II, and ZZ contributed to conception of the review article. CT wrote the first draft and sections of the manuscript. All authors contributed to manuscript revision, read and approved the submitted version.

## Conflict of Interest Statement

The authors declare that the research was conducted in the absence of any commercial or financial relationships that could be construed as a potential conflict of interest.

## Abbreviations

DMAPP: dimethylallyl pyrophosphate
DXP: 1-deoxy-D-xylulose-5-phosphate
FPP: farnesyl pyrophosphate
FPS: FPP synthase
FTPEC: flindersia-type 2-(2-phenylethyl) chromone
HMGR: 3-hydroxy-3-methylglutaryl-CoA
IPP: isopentenyl pyrophosphate
JA: Jasmonic acid
MAPK: mitogen-activated protein kinase
MAS: marker-assisted selection
MeJA: methyl jasmonate
MEP: methylerythritol phosphate
MVA: mevalonic acid
NADPH: dihydronicotinamide-adenine dinucleotide phosphate
OMT: *O*-methyltransferase
P450: cytochrome P450 dependent mono-oxygenase
PAMP: pathogen-associated molecular pattern
PCD: programmed cell death
PEC: 2-(2-phenylethyl) chromone
PKs: polyketide synthases
POR: NADPH-dependent cytochrome P450 oxidoreductase
SA: salicylic acid
SesTP: sesquiterpene synthase
SMRT: single molecule real time sequencing technology
TF: transcription factor.

## References

[B1] Abdin M. J. (2014). The agar wood industry: yet to utilize in Bangladesh. *Int. J. Econ. Manag. Sci.* 3 163–166. 10.2139/ssrn.2430055

[B2] Akter S. Islam M. T. Zulkefeli M. Khan S. I. (2013). Agarwood production - a multidisciplinary field to be explored in Bangladesh. *Int. J. Pharm. Life Sci.* 2 22–32. 10.3329/ijpls.v2i1.15132

[B3] Ambawat S. Sharma P. Yadav N. R. Yadav R. C. (2013). MYB transcription factor genes as regulators for plant responses: an overview. *Physiol. Mol. Biol. Plants.* 19 307–321. 10.1007/s12298-013-0179-1. 24431500 PMC3715649

[B4] Antonopoulou M. Compton J. Perry L. S. Al-Mubarak R. (2010). ”The trade and use of agarwood (oudh) in the United Arab Emirates,” in: *TRAFFIC Southeast Asia.* (Geneva: The CITES secretariat).

[B5] Azren P. D. Lee S. Y. Emang D. Mohamed R. (2018). History and perspectives of induction technology for agarwood production from cultivated Aquilaria in Asia: a review. *J. For. Res.* 30 1–11. 10.1007/s11676-018-0627-4.

[B6] Azzarina A. B. Mohamed R. Lee S. Y. Nazre M. (2016). Temporal and spatial expression of terpene synthase genes associated with agarwood formation in *Aquilaria malaccensis* Lam. *N. Z. J. For. Sci.* 46:12 10.1186/s40490-016-0068-9.

[B7] Blanchette R. Heuveling V. B. H. (2009). *Cultivated Agarwood.* U.S. Patent No. 7638145. Minnesota: University of Minnesota.

[B8] Chen H. Q. Wei J. H. Yang J. S. Zhang Z. Yang Y. Gao Z. H. (2012). Chemical constituents of agarwood originating from the endemic genus Aquilaria plants. *Chem. Biodivers.* 9 236–250. 10.1002/cbdv.201100077. 22344902

[B9] Chhipa H. Kaushik N. (2017). Fungal and bacterial diversity isolated from *Aquilaria malaccensis* tree and soil, induces agarospirol formation within 3 months after artificial infection. *Front. Microbiol.* 8:1286. 10.3389/fmicb.2017.01286. 28747900 PMC5507295

[B10] Convention on International Trade in Endangered Species [CITES] (2004). ”Convention on international trade in endangered species of wild fauna and flora. Consideration of proposals for amendment of appendices-I and -II *Aquilaria* spp. and Gyrinops spp.,” in *Proceedings of the Thirteenth Meeting of the Conference of the Parties.* Bangkok.

[B11] Cui J. Guo S. Fu S. Xiao P. Wang M. (2013). Effects of inoculating fungi on agilawood formation in *Aquilaria sinensis*. *Chin. Sci. Bull.* 58 3280–3287. 10.1007/s11434-013-5856-5.

[B12] Dong L. Jongedijk E. Bouwmeester H. Van Der Krol A. (2015). Monoterpene biosynthesis potential of plant subcellular compartments. *New Phytol.* 209 679–690. 10.1111/nph.13629. 26356766

[B13] Fazila K. N. Halim K. H. K. (2012). Effects of soaking on yield and quality of agarwood oil. *J. Trop. For. Sci.* 24 557–564.

[B14] Gaffe J. Bru J. P. Causse M. Vidal A. Stamitti-Bert L. Carde J. P. (2000). LEFPS1, a tomato farnesyl pyrophosphate gene highly expressed during early fruit development. *Plant Physiol.* 123 1351–1362. 10.1104/pp.123.4.1351 10938353 PMC59093

[B15] Gao X. Xie M. Liu S. Guo X. Chen X. Zhong Z. (2014). Chromatographic fingerprint analysis of metabolites in natural and artificial agarwood using gas chromatography-mass spectrometry combined with chemometric methods. *J. Chromatogr. B Analyt. Technol. Biomed. Life Sci.* 967 264–273. 10.1016/j.jchromb.2014.07.039. 25129412

[B16] Gao Z. H. Yang Y. Zhang Z. Zhao W. T. Meng H. Jin Y. (2014). Profiling of microRNAs under wound treatment in *Aquilaria sinensis* to identify possible microRNAs involved in agarwood formation. *Int. J. Biol. Sci.* 10 500–510. 10.7150/ijbs.8065. 24795531 PMC4007363

[B17] Gao Z. H. Wei J. H. Yang Y. Zhang Z. Xiong H. Y. Zhao W. T. (2012a). Identification of conserved and novel microRNAs in *Aquilaria sinensis* based on small RNA sequencing and transcriptome sequence data. *Gene* 505 167–175. 10.1016/j.gene.2012.03.072. 22521867

[B18] Gao Z. H. Wei J. H. Yang Y. Zhang Z. Zhao W. T. (2012b). Selection and validation of reference genes for studying stress-related agarwood formation of *Aquilaria sinensis*. *Plant Cell Rep.* 31 1759–1768. 10.1007/s00299-012-1289-x. 22678434

[B19] Goel S. S. Makrandi J. K. (2006). Synthesis of 2-(2-phenylethyl)chromones. *Indian J. Chem.* 45B, 535–536. 10.1002/chin.200623140

[B20] Gong B. Yan Y. Wen D. Shi Q. (2017). Hydrogen peroxide produced by NADPH oxidase: a novel downstream signaling pathway in melatonin-induced stress tolerance in Solanum lycopersicum. *Physiol. Plant.* 160 396–409. 10.1111/ppl.12581. 28464254

[B21] Hashim Y. Ismail N. Abbas P. (2014). Analysis of chemical compounds of agarwood oil from different species by gas chromatography mass spectrometry (GCMS). *IIUM Eng. J.* 15 55–60. 10.31436/iiumej.v15i1.469. 30154355

[B22] Hashim Y. Z. Kerr P. G. Abbas P. Mohd Salleh H. (2016). Aquilaria spp. (agarwood) as source of health beneficial compounds: a review of traditional use, phytochemistry and pharmacology. *J. Ethnopharmacol.* 189 331–360. 10.1016/j.jep.2016.06.055. 27343768

[B23] Hong G. J. Xue X. Y. Mao Y. B. Wang L. J. Chen X. Y. (2012). Arabidopsis MYC2 interacts with DELLA proteins in regulating sesquiterpene synthase gene expression. *Plant Cell* 24 2635–2648. 10.1105/tpc.112.098749. 22669881 PMC3406894

[B24] Hung K. T. Hsu Y. T. Kao C. H. (2006). Hydrogen peroxide is involved in methyl jasmonate-induced senescence of rice leaves. *Physiol. Plant.* 127 293–303. 10.1111/j.1399-3054.2006.00662.x.

[B25] Ibrahim S. R. (2014). New chromone and triglyceride from Cucumis melo seeds. *Nat. Prod. Commun.* 9 205–208. 24689290

[B26] Ibrahim S. R. Mohamed G. A. (2015). Natural occurring 2-(2-phenylethyl) chromones, structure elucidation and biological activities. *Nat. Prod. Res.* 29 1489–1520. 10.1080/14786419.2014.991323. 25529202

[B27] Ishihara M. Tsuneya T. Uneyama K. (1993). Components of the volatile concentrate of agarwood. *J. Essent. Oil Res.* 5:3 10.1080/10412905.1993.9698221.

[B28] Ito M. Okimoto K. I. Yagura T. Honda G. Kiuchi F. Shimada Y. (2005). Induction of sesquiterpenoid production by methyl jasmonate in *Aquilaria sinensis* cell suspension culture. *J. Essent. Oil Res.* 17 175–180. 10.1080/10412905.2005.9698867

[B29] Jayachandran K. Sekar I. Parthiban K. T. Amirtham D. Suresh K. K. (2014). Analysis of different grades of agarwood (*Aquilaria malaccensis* Lamk.) oil through GC-MS. *Indian J. Nat. Prod. Resour.* 5 44–47.

[B30] Jong P. L. Pascale T. Rozi M. (2014). Gas chromatography-mass spectrometry analysis of agarwood extracts from mature and juvenile *Aquilaria malaccensis*. *Int. J. Agric. Biol.* 16 644–648.

[B31] Kalita J. (2015). Association of *Zeuzera conferta* Walker on agarwood formation in *Aquilaria malaccensis* Lamk. *Asian J. Plant Sci. Res.* 5 4–9.

[B32] Kenmotsu Y. Ogita S. Katoh Y. Yamamura Y. Takao Y. Tatsuo Y. (2011). Methyl jasmonate-induced enhancement of expression activity of Am-FaPS-1, a putative farnesyl diphosphate synthase gene from *Aquilaria microcarpa*. *J. Nat. Med.* 65 194–197. 10.1007/s11418-010-0451-4. 20686864

[B33] Khadem S. Marles R. J. (2011). Chromone and flavonoid alkaloids: occurrence and bioactivity. *Molecules* 17 191–206. 10.3390/molecules17010191. 22202807 PMC6268529

[B34] Kumeta Y. Ito M. (2010). Characterization of δ-guaiene synthases from cultured cells of Aquilaria, responsible for the formation of the sesquiterpenes in agarwood. *Plant Physiol.* 154 1998–2007. 10.1104/pp.110.161828. 20959422 PMC2996018

[B35] Lee S.Y. Mohamed R. (2016). ”The origin and domestication of Aquilaria, an important agarwood-producing genus,” in *Agarwood: Science Behind the Fragrance*, ed. Mohamed R. (Berlin: Springer Singapore), 1–20. 10.1007/978-981-10-0833-7_1

[B36] Li Y. Zhang Q. Zhang J. Wu L. Qi Y. Zhou J. M. (2010). Identification of microRNAs involved in pathogen-associated molecular pattern-triggered plant innate immunity. *Plant Physiol.* 152 2222–2231. 10.1104/pp.109.151803. 20164210 PMC2850012

[B37] Liao G. Dong W. -H. Yang J. -L. Li W. Wang J. Mei W. -L. (2018). Monitoring the chemical profile in agarwood formation within one year and speculating on the biosynthesis of 2-(2-phenylethyl) chromones. *Molecules* 23:1261. 10.3390/molecules23061261. 29799457 PMC6100365

[B38] Liao Y. Wei J. Xu Y. Zhang Z. (2015). Cloning, expression and characterization of COI1 gene (AsCOI1) from *Aquilaria sinensis* (Lour.) Gilg. *Acta. Pharm. Sin. B.* 5 473–481. 10.1016/j.apsb.2015.05.009. 26579478 PMC4629437

[B39] Liu J. Xu Y. Zhang Z. Wei J. (2015). Hydrogen peroxide promotes programmed cell death and salicylic acid accumulation during the induced production of sesquiterpenes in cultured cell suspensions of *Aquilaria sinensis*. *Funct. Plant Biol.* 42 337–346. 10.1071/FP14189. 32480678

[B40] Liu X. M. Tao T. T. Meng X. X. Zhang W. W. Chang J. Xu F. (2017). Cloning and expression analysis of a farnesyl diphosphate synthase (FPPS) gene from Chamaemelum nobile. *Not. Bot. Horti Agrobo. Cluj-Na.* 45:2 10.15835/nbha45210858.

[B41] Liu X. Zhang B.-F. Yang L. Chou G.-X. Wang Z.-T. (2013). Two new chromones and a new flavone glycoside from imperata cylindrica. *Chin. J. Nat. Med.* 11 77–80. 10.1016/S1875-5364(13)60012-6.

[B42] Liu Y. Chen H. Yang Y. Zhang Z. Wei J. Meng H. (2013). Whole-tree agarwood-inducing technique: an efficient novel technique for producing high-quality agarwood in cultivated *Aquilaria sinensis* trees. *Molecules* 18 3086–3106. 10.3390/molecules18033086. 23470337 PMC6270329

[B43] Liu Y. Y. Wei J. H. Gao Z. H. Zhang Z. Lyu J. C. (2017). A review of quality assessment and grading for agarwood. *Chin. Herb. Med.* 9 22–30. 10.1016/S1674-6384(17)60072-8.

[B44] López-Sampson A. Page T. (2018). History of use and trade of agarwood. *Econ. Bot.* 72 107–129. 10.1007/s12231-018-9408-4.

[B45] Ma D. Pu G. Lei C. Ma L. Wang H. Guo Y. (2009). Isolation and characterization of AaWRKY1, an Artemisia annua transcription factor that regulates the amorpha-4,11-diene synthase gene, a key gene of artemisinin biosynthesis. *Plant Cell Physiol.* 50 2146–2161. 10.1093/pcp/pcp149. 19880398

[B46] Mei W. -L. Yang D. -L. Wang H. Yang J. -L. Zeng Y. -B. Guo Z. -K. (2013). Characterization and determination of 2-(2-Phenylethyl)chromones in agarwood by GC-MS. *Molecules* 18 12324–12345. 10.3390/molecules181012324. 24108398 PMC6269921

[B47] Mochida K. Shinozaki K. (2011). Advances in omics and bioinformatics tools for systems analyses of plant functions. *Plant Cell Physiol.* 52 2017–2038. 10.1093/pcp/pcr153. 22156726 PMC3233218

[B48] Mohamed R. Jong P. L. Kamziah A. K. (2014). Fungal inoculation induces agarwood in young *Aquilaria malaccensis* trees in the nursery. *J. For. Res.* 25 201–204. 10.1007/s11676-013-0395-0.

[B49] Mohamed R. Jong P. L. Zali M. S. (2010). Fungal diversity in wounded stems of *Aquilaria malaccensis*. *Fungal Divers.* 43 67–74. 10.1007/s13225-010-0039-z.

[B50] Naef R. (2011). The volatile and semi-volatile constituents of agarwood, the infected heartwood of Aquilaria species: a review. *Flavour Fragr. J.* 26 73–87. 10.1002/ffj.2034.

[B51] Nagegowda D. A. (2010). Plant volatile terpenoid metabolism: biosynthetic genes, transcriptional regulation and subcellular compartmentation. *FEBS Lett.* 584 2965–2973. 10.1016/j.febslet.2010.05.045. 20553718

[B52] Orozco-Cardenas M. L. Narvaez-Vasquez J. Ryan C. A. (2001). Hydrogen peroxide acts as a second messenger for the induction of defense genes in tomato plants in response to wounding, systemin, and methyl jasmonate. *Plant Cell* 13 179–191. 10.1105/tpc.13.1.179 11158538 PMC102208

[B53] Pasaribu G. T. Waluyo T. K. Pari G. (2015). Analysis of chemical compounds distinguisher for agarwood qualities. *Indonesian J. For. Res.* 2:7 10.20886/ijfr.2015.2.1.1-7

[B54] Pateraki I. Heskes A. Hamberger B. (2015). Cytochromes P450 for terpene functionalisation and metabolic engineering. *Adv. Biochem. Eng. Biotechnol.* 148: 207-239. 10.1007/10_2014_301 25636487

[B55] Phukan U. J. Jeena G. S. Shukla R. K. (2016). WRKY transcription factors: molecular regulation and stress responses in plants. *Front. Plant Sci.* 7:760. 10.3389/fpls.2016.00760. 27375634 PMC4891567

[B56] Rasool S. Mohamed R. (2016). ”Understanding agarwood formation and its challenges,” in *Agarwood: Science Behind the Fragrance*, ed. Mohamed R. (Berlin: Springer), 39–56. 10.1007/978-981-10-0833-7_3

[B57] Reis J. Gaspar A. Milhazes N. Borges F. (2017). Chromone as a privileged scaffold in drug discovery: recent advances. *J. Med. Chem.* 60 7941–7957. 10.1021/acs.jmedchem.6b01720. 28537720

[B58] Rhoads A. Au K. F. (2015). PacBio sequencing and its applications. *Genom. Proteom. Bioinform.* 13 278–289. 10.1016/j.gpb.2015.08.002. 26542840 PMC4678779

[B59] Rohmer M. (1999). The discovery of a mevalonate-independent pathway for isoprenoid biosynthesis in bacteria, algae and higher plants. *Nat. Prod. Rep.* 16 565–574. 10.1039/a709175c 10584331

[B60] Sangareswari Nagajothi M. Thangamuthu Parthiban K. Umesh Kanna S. Karthiba L. Saravanakumar D. (2016). Fungal microbes associated with agarwood formation. *Am. J. Plant Sci.* 7 1445–1452. 10.4236/ajps.2016.710138

[B61] Schmiesing A. Emonet A. Gouhier-Darimont C. Reymond P. (2016). Arabidopsis MYC transcription factors are the target of hormonal salicylic acid/jasmonic acid cross talk in response to Pieris brassicae egg extract. *Plant Physiol.* 170:2432. 10.1104/pp.16.00031 26884488 PMC4825139

[B62] Shao H. Mei W. -L. Dong W. -H. Gai C. -J. Li W. Zhu G. -P. (2016). 2-(2-phenylethyl)chromone derivatives of agarwood originating from *Gyrinops salicifolia*. *Molecules* 21:E1313. 10.3390/molecules21101313. 27706109 PMC6273548

[B63] Shimada Y. Tominaga T. Kiyosawa S. (1986). Studies on the agalwood.(Jinko). IV: correlation between the grading of agalwood on the market and the chromone derivatives. *Yakugaku Zasshi J. Pharm. Soc. Jpn.* 106 391–397. 10.1248/yakushi1947.106.5_391

[B64] Shimada Y. Tominaga T. Konishi T. Kiyosawa S. (1982). Studies on the agarwood (Jinko). I. structures of 2-(2-phenylethyl) chromone derivatives. *Chem. Pharm. Bull.* 30 3791–3795. 10.1248/cpb.30.3791.

[B65] Siburian R. H. Siregar U. J. Siregar I. Z. Santoso E. (2015). Identification of morphological characters of *Aquilaria microcarpa* in the interaction with *Fusarium solani*. *Int. J. Sci. Basic Appl. Res.* 20:119–128.

[B66] Singh B. Sharma R. A. (2015). Plant terpenes: defense responses, phylogenetic analysis, regulation and clinical applications. *3 Biotech.* 5 129–151. 10.1007/s13205-014-0220-2. 28324581 PMC4362742

[B67] Sinha A. K. Jaggi M. Raghuram B. Tuteja N. (2011). Mitogen-activated protein kinase signaling in plants under abiotic stress. *Plant Signal. Behav.* 6 196–203. 10.4161/psb.6.2.14701. 21512321 PMC3121978

[B68] Subasinghe S. M. C. U. P. Hettiarachchi D. S. (2015). Characterisation of agarwood type resin of *Gyrinops walla* Gaertn growing in selected populations in Sri Lanka. *Ind. Crops Prod.* 69 76–79. 10.1016/j.indcrop.2015.01.060.

[B69] Subasinghe U. Hettiarachchi D. (2013). Agarwood resin production and resin quality of *Gyrinops walla* Gaertn. *Int. J. Agr. Sci.* 3 357–362.

[B70] Tawfik H. A. Ewies E. F. El-Hamouly W. S. (2014). Synthesis of chromones and their applications during the last ten years. *Int. J. Res. Pharm. Chem.* 4 1046–1085.

[B71] Tuteja N. Mahajan S. (2007). Calcium signaling network in plants: an overview. *Plant Signal. Behav.* 2 79–85. 10.4161/psb.2.2.4176 19516972 PMC2633903

[B72] Van Thanh L. Van Do T. Son N. H. Sato T. Kozan O. (2015). Impacts of biological, chemical and mechanical treatments on sesquiterpene content in stems of planted *Aquilaria crassna* trees. *Agroforest. Syst.* 89 973–981. 10.1007/s10457-015-9829-3.

[B73] Wang S. L. Hwang T. L. Chung M. I. Sung P. J. Shu C. W. Cheng M. J. (2015). New flavones, a 2-(2-phenylethyl)-4H-chromen-4-one derivative, and anti-inflammatory constituents from the stem barks of *Aquilaria sinensis*. *Molecules* 20 20912–20925. 10.3390/molecules201119736. 26610457 PMC6332152

[B74] Wang T. Li L. F. Zhang K. Zhang W. Y. Pei Y. H. (2001). New 2-(2-phenylethyl) chromones from *Bothriochloa ischaemum*. *J. Asian Nat. Prod. Res.* 3 145–149. 10.1080/10286020108041382. 11407814

[B75] Wang X. Gao B. Liu X. Dong X. Zhang Z. Fan H. (2016). Salinity stress induces the production of 2-(2-phenylethyl)chromones and regulates novel classes of responsive genes involved in signal transduction in *Aquilaria sinensis* calli. *BMC Plant Biol.* 16:119. 10.1186/s12870-016-0803-7. 27230436 PMC4881210

[B76] Wu B. Lee J. Lim C. Dong Jia S. Won Kwon S. Seo Hwang G. (2012a). Sesquiterpenoids and 2-(2-phenylethyl)-4H-chromen-4-one ( = 2-(2-phenylethyl)-4H-1-benzopyran-4-one) derivatives from *Aquilaria malaccensis* agarwood. *Helv. Chim. Acta* 95 636–642. 10.1002/hlca.201100409

[B77] Wu B. Won Kwon S. Seo Hwang G. Hill Park J. (2012b). Eight new 2-(2-phenylethyl)chromone ( = 2-(2-Phenylethyl)-4H-1-benzopyran-4-one) derivatives from *Aquilaria malaccensis* agarwood. *Helv. Chim. Acta* 95 1657–1665. 10.1002/hlca.201200069.

[B78] Wu Z. Q. Liu S. Li J. F. Li M. C. Du H. F. Qi L. K. (2017). Analysis of gene expression and quality of agarwood using Agar-bit in *Aquilaria sinensis*. *J. Trop. For. Sci.* 29 380–388. 10.26525/jtfs2017.29.3.380388

[B79] Xu C. Liu R. Zhang Q. Chen X. Qian Y. Fang W. (2017). The diversification of evolutionarily conserved MAPK cascades correlates with the evolution of fungal species and development of lifestyles. *Genome Biol. Evol.* 9 311–322. 10.1093/gbe/evw051. 26957028 PMC5381651

[B80] Xu Y. -H. Liao Y. -C. Zhang Z. Liu J. Sun P. -W. Gao Z. -H. (2016). Jasmonic acid is a crucial signal transducer in heat shock induced sesquiterpene formation in *Aquilaria sinensis*. *Sci. Rep.* 6:21843. 10.1038/srep21843 26902148 PMC4763180

[B81] Xu Y. Zhang Z. Wang M. Wei J. Chen H. Gao Z. (2013). Identification of genes related to agarwood formation: transcriptome analysis of healthy and wounded tissues of *Aquilaria sinensis*. *BMC Genomics* 14:227. 10.1186/1471-2164-14-227. 23565705 PMC3635961

[B82] Xu Y. H. Liao Y. C. Lv F. F. Zhang Z. Sun P. W. Gao Z. H. (2017). Transcription factor AsMYC2 controls the jasmonate-responsive expression of ASS1 regulating sesquiterpene biosynthesis in *Aquilaria sinensis* (Lour.) Gilg. *Plant Cell Physiol.* 58 1924–1933. 10.1093/pcp/pcx122. 29016977

[B83] Xu Y. H. Wang J. W. Wang S. Wang J. Y. Chen X. Y. (2004). Characterization of GaWRKY1, a cotton transcription factor that regulates the sesquiterpene synthase gene (+)-delta-cadinene synthase-A. *Plant Physiol.* 135 507–515. 10.1104/pp.104.038612. 15133151 PMC429402

[B84] Yang D. L. Wang H. Guo Z. K. Dong W. H. Mei W. L. Dai H. F. (2014a). A new 2-(2-phenylethyl)chromone derivative in Chinese agarwood ’Qi-Nan’ from *Aquilaria sinensis*. *J. Asian Nat. Prod. Res.* 16 770–776. 10.1080/10286020.2014.896342. 24646200

[B85] Yang D. -L. Wang H. Guo Z. -K. Li W. Mei W. -L. Dai H. -F. (2014b). Fragrant agarofuran and eremophilane sesquiterpenes in agarwood ‘Qi-Nan’ from *Aquilaria sinensis*. *Phytochem. Lett.* 8 121–125. 10.1016/j.phytol.2014.03.003.

[B86] Yang L. Qiao L. Xie D. Yuan Y. Chen N. Dai J. (2012). 2-(2-phenylethyl)chromones from Chinese eaglewood. *Phytochem.* 76 92–97. 10.1016/j.phytochem.2011.11.017. 22243963

[B87] Yang M. Fu H. Liang Y. Huang H. Zhao B. Xie C. (2014c). Modified transfusion devices, inducer, and procedure for agarwood-inducing by infusion technique. *J. Chem. Pharm. Res.* 6 2566–2571.

[B88] Yang X. Wei J. H. Liu J. Xu Y. H. (2013). Cloning and expression analysis of farnesyl pyrophosphate synthase from *Aquilaria sinensis*. *Zhongguo Zhong Yao Za Zhi.* 38 3251–3255. 24422386

[B89] Ye W. He X. Wu H. Wang L. Zhang W. Fan Y. (2018). Identification and characterization of a novel sesquiterpene synthase from *Aquilaria sinensis*: an important gene for agarwood formation. *Int. J. Biol. Macromol.* 108 884–892. 10.1016/j.ijbiomac.2017.10.183. 29102787

[B90] Ye W. Wu H. He X. Wang L. Zhang W. Li H. (2016). Transcriptome sequencing of chemically induced Aquilaria sinensis to identify genes related to agarwood formation. *PLoS One* 11:e0155505. 10.1371/journal.pone.0155505. 27182594 PMC4868263

[B91] Yoswathana N. (2013). Extraction of agarwood (*Aquilaria crassna*) oil by using supercritical carbon dioxide extraction and enzyme pretreatment on hydrodistillation. *J. Food Agric. Environ.* 11 1055–1059.

[B92] Zhang X. L. Liu Y. Y. Wei J. H. Yang Y. Zhang Z. Huang J. Q. (2012). Production of high-quality agarwood in *Aquilaria sinensis* trees via whole-tree agarwood-induction technology. *Chin. Chem. Lett.* 23 727–730. 10.1016/j.cclet.2012.04.019.

